# Mucinous cystadenoma of the renal parenchyma presenting as a Bosniak IIF complex renal cyst

**DOI:** 10.1002/iju5.12576

**Published:** 2023-01-18

**Authors:** Takanori Nate, Koji Hatano, Taigo Kato, Atsunari Kawashima, Toyofumi Abe, Shinichiro Fukuhara, Motohide Uemura, Hiroshi Kiuchi, Ryoichi Imamura, Norio Nonomura

**Affiliations:** ^1^ Department of Urology Osaka University Graduate School of Medicine Suita Japan

**Keywords:** cyst, cystadenoma, kidney, mucin, neoplasm

## Abstract

**Introduction:**

A primary retroperitoneal mucinous cystadenoma should be surgically resected because of the risk of malignant transformation. However, mucinous cystadenoma of the renal parenchyma is very rare, and preoperative imaging mimics complicated renal cysts.

**Case presentation:**

A 72‐year‐old woman presented with a right renal mass on computed tomography that was followed up as a Bosniak IIF complicated renal cyst. One year later, the right renal mass gradually increased in size. Abdominal computed tomography showed an 11 × 10 cm mass in the right kidney. A laparoscopic right nephrectomy was performed because cystic carcinoma of the kidney was suspected. Pathologically, the tumor was diagnosed as mucinous cystadenoma of the renal parenchyma. Eighteen months after resection, the disease has not recurred.

**Conclusion:**

Here, we experienced a case of a renal mucinous cystadenoma as a slowly enlarging Bosniak IIF complex renal cyst.

**Abbreviations & Acronyms**
CT: computed tomography
CN: cystic nephroma
CK: cytokeratin
ER: estrogen receptor
MRI: magnetic resonance imaging
MEST: mixed epithelial and stromal tumor
T1WI: T1‐weighted imaging
T2WI: T2‐weighted imaging

**Keynote message**

A renal mucinous cystadenoma is rare but should be surgically resected because of the risk of malignant transformation. However, it is difficult to correctly diagnose the renal mucinous cystadenoma on imaging. An enlarging Bosniak IIF renal cyst may be a differential diagnosis for a mucinous cystadenoma.

## Introduction

Mucinous cystadenoma of the renal parenchyma is very rare,^1^, ^2^, ^3^, ^4^ and its origin and clinicopathological features have not been clarified. However, a primary retroperitoneal mucinous cystadenoma should be surgically resected because of the risk of malignant transformation.^5^, ^6^, ^7^, ^8^ We report our experience with a renal mucinous cystadenoma in the form of a Bosniak IIF complex renal cyst.

## Case presentation

**Fig. 1:**
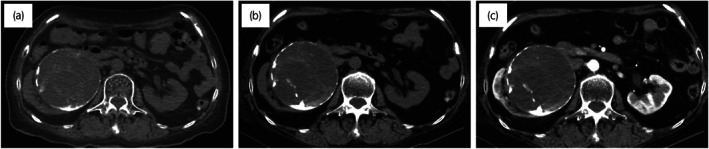
Abdominal CT. (a) Simple CT 1 year before surgery shows a 9 cm mass in the right kidney with calcification in the septum. (b) Simple CT and (c) contrast‐enhanced CT before surgery. CT shows an 11 × 10 cm mass in the right kidney, but the contrast effect is low.

**Fig. 2:**
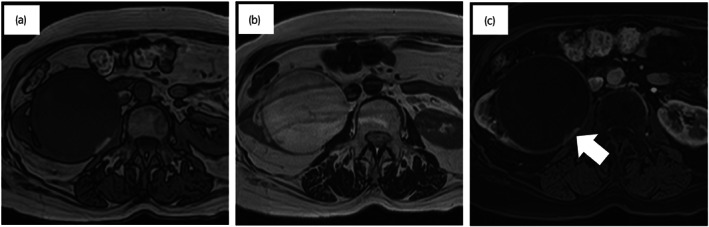
Abdominal MRI. (a) T1WI, (b) T2WI, and (c) dynamic contrast‐enhanced MRI. The arrow indicates an enhanced small nodule within the capsule of the tumor.

**Fig. 3:**
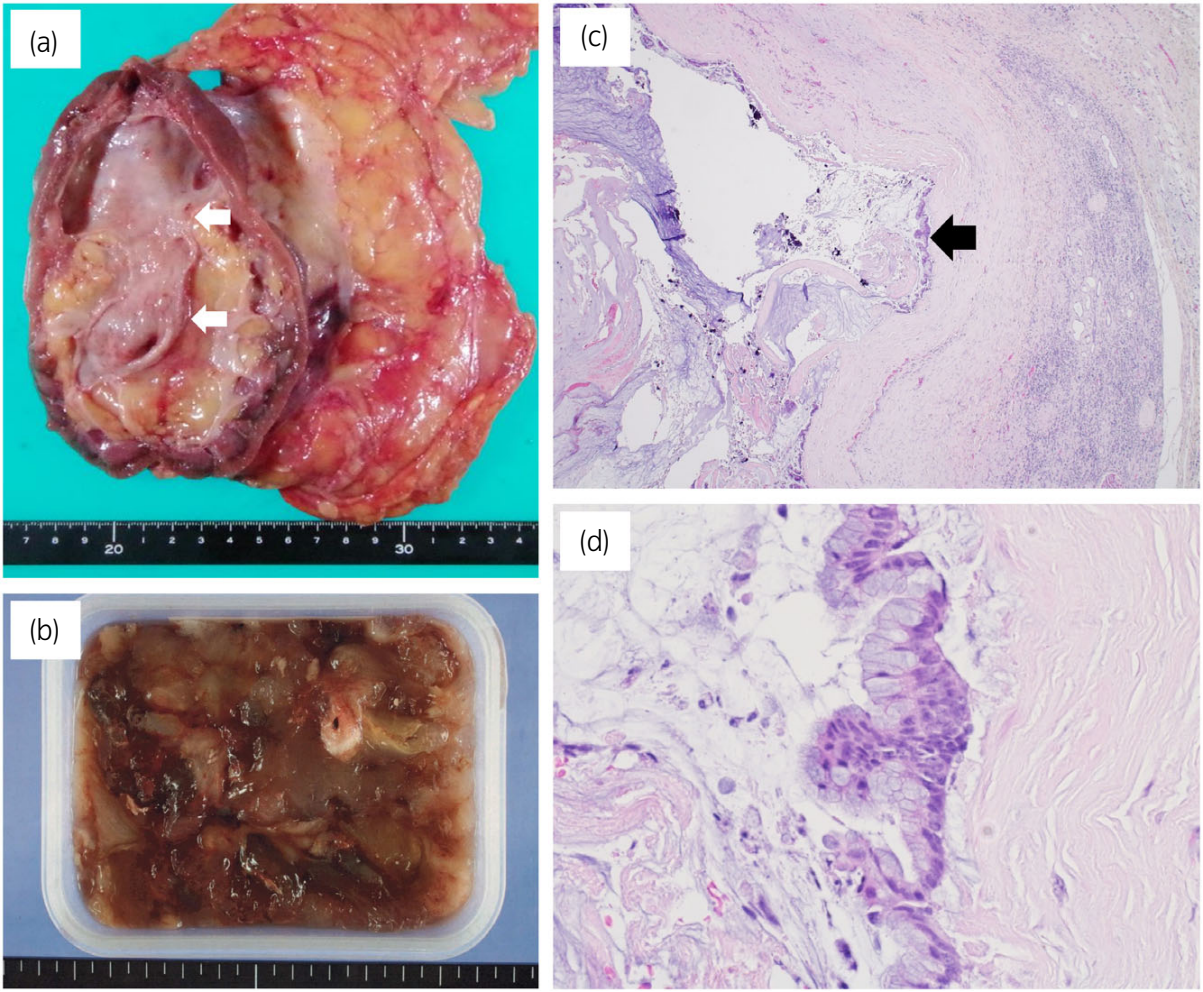
Pathological findings of the tumor. (a) Macroscopic image of the right renal tumor. The arrows indicate a unilocular cystic tumor. (b) A large amount of mucus is present inside the tumor. Microscopic image of the tumor with (c) weak magnification and (d) strong magnification (hematoxylin and eosin staining). The cystic lesion had a mucinous epithelial lining with mild‐to‐moderate atypia. The arrow indicates the mucinous epithelial.

A 72‐year‐old woman with hypertension and hyperlipidemia had a right renal mass 9 cm in size that was noted on CT for close examination of breast cancer; however, this was followed up as a Bosniak IIF complicated renal cyst (Fig. 1a). One year later, it was found that the renal mass had gradually increased in size. Abdominal CT showed an 11 × 10 cm mass in the right kidney with low contrast effect and calcification in the septum; the mass presented as a Bosniak IIF renal cyst (Fig. 1b,c). The appendix was normal. Abdominal MRI showed low signal on T1WI and high signal on T2WI, with an enhanced small nodule within the capsule, and the mass was determined to be Bosniak III (Fig. 2). Resection was the surgical choice because cystic carcinoma of the kidney was suspected. Preoperative renal function was Cr 0.67 mg/dL and eGFR 65.1. A laparoscopic right nephrectomy was performed. The nephrectomy specimen showed the formation of a unilocular cystic lesion with a large amount of mucus inside (Fig. 3a,b). The cystic lesion had no traffic with the renal pelvis. Pathological findings revealed that the cystic lesion had mucinous epithelial lining with mild‐to‐moderate atypia, but the stroma had no atypia (Fig. 3c,d). Immunohistochemistry revealed that epithelial cells were positive for CDX2, CK7, and CK20. Both epithelial and stromal cells were negative for PAX8, GATA3, CD10, and ER. The tumor was diagnosed as mucinous cystadenoma of the renal parenchyma. Three months after surgery, a CT scan showed an enlarged appendix (1 × 5 cm diameter). An appendiceal mucinous tumor was suspected. Thus, laparoscopic ileocecal resection was performed, and pathological findings revealed appendicitis with reactive mucus production. The patient experienced no recurrence 18 months after resection of the renal mass.

## Discussion

To date, 23 cases of renal mucinous cystadenoma have been reported worldwide, and these are divided into renal pelvis and parenchymal tumors.^1^, ^2^, ^3^, ^4^ Among them, renal parenchymal tumor has only been reported in the English literature in four cases, including the present case, and two of them originated from horseshoe kidneys (Table 1). Mucinous tumors of renal pelvis are associated with a history of stones and pyelonephritis, and chronic irritation of the transitional epithelium is considered a possible factor in their development.^9^ Mitome *et al*. reported a mucinous tumor originating from a sequestered segment of the renal pelvic epithelium in the renal parenchyma in a case of horseshoe kidney.^3^ The origin of mucinous cystadenoma of the renal parenchyma is still unknown but is thought to be due, in part, to stray migration of the transitional epithelium during development.

**Table 1:** Case reports of mucinous cystadenoma of the renal parenchyma

Author	Age	Sex	Origin	Size (cm)	Bosniak classification	Surgery
Akan *et al*.^1^	27	Female	Right (horseshoe kidney)	12	I	Cyst excision
Mitome *et al*.^3^	45	Male	Left (horseshoe kidney)	16.5	IIF	Heminephrectomy
Kalantari *et al*.^4^	66	Male	Left	6.4	IIF	Nephrectomy
Present case	72	Female	Right	11	IIF	Nephrectomy

CN and MEST are both benign lesions of the kidney with biphasic epithelial and stromal components. According to the 2016 World Health Organization classification, MEST encompasses a spectrum of tumors ranging from predominantly cystic tumors (CN) to tumors with diverse solid components (MEST).^10^ In the majority of CN and MEST, ER and progesterone receptor expression is found in the stromal cells.^11^, ^12^ Chu PG *et al*., reported a mucinous borderline tumor arising from MEST of the kidney, with positive ER in stromal cells.^13^ Although the stroma of the tumor had no atypia in this case and the ER was negative, there are limitations in completely differentiating MEST from renal mucinous cystadenoma.

The standard treatment for renal mucinous cystadenoma is complete resection because of the risk of malignant transformation.^5^, ^6^ However, it is difficult to correctly diagnose renal mucinous cystadenoma and distinguish it from a complicated cyst on imaging. The Bosniak classification is widely used for the diagnosis of cystic renal masses in order to identify malignant tumors.^14^ In the systemic review by Schoots *et al*., stable Bosniak IIF cysts showed a malignancy rate of less than 1% during radiological follow‐up.^15^ However, Bosniak IIF cysts, which showed reclassification to the Bosniak III/IV category during radiological follow‐up (12%), showed malignancy in 85% of cases, comparable to that for Bosniak IV cysts.^15^ The Bosniak classification of mucinous cystadenoma of renal parenchyma (I to IIF) for the four cases is shown in Table 1. In the present case, the renal mass presented as a Bosniak IIF cyst with a tendency of slow growth. Thus, an enlarging Bosniak IIF tumor, although infrequent, may be a differential diagnosis for mucinous tumors.

Mucinous tumors predominantly occur in the appendix and ovaries.^16^, ^17^, ^18^ The appendiceal mucinous neoplasia can cause pseudomyxoma peritonei and metastasize to the ovary.^16^, ^17^, ^18^ Because it is often difficult to distinguish primary ovarian from metastatic mucinous tumors of the appendix, pathologists insist that the diagnosis of primary mucinous ovarian neoplasm requires consideration and exclusion of metastases from other gastrointestinal carcinomas.^17^ It is considered unnecessary to perform an appendectomy if the appendix is grossly normal during surgeries for mucinous ovarian tumor.^16^, ^17^ In this case, we performed careful follow‐up after the surgery of renal mucinous cystadenoma and noted an enlarged appendix; however, the pathology showed appendicitis, and metastasis was thus ruled out. To date, there have been no reports of renal mucinous cystadenoma metastasizing to other organs, nor have any mucinous tumors metastasized to the kidney. Thus, the relationship between renal mucinous cystadenoma and mucinous tumors of the appendix and ovary remains unclear.

## Conclusion

Here, we reported a case of mucinous cystadenoma of the renal parenchyma presenting as a slowly enlarging Bosniak IIF complex renal cyst.

## Author contributions

Takanori Nate: Data curation; writing – original draft. Koji Hatano: Conceptualization; data curation; funding acquisition; writing – original draft; writing – review and editing. Taigo Kato: Data curation. Atsunari Kawashima: Data curation. Toyofumi Abe: Data curation; methodology. Shinichiro Fukuhara: Data curation. Motohide Uemura: Data curation. Hiroshi Kiuchi: Data curation. Ryoichi Imamura: Supervision. Norio Nonomura: Supervision.

## Conflict of interest

The authors declare no conflict of interest.

## Approval of the research protocol by an Institutional Reviewer Board

No 13397‐20.

## Informed consent

All human subjects provided written informed consent with guarantees of confidentiality.

## Registry and the Registration No. of the study/trial

Not applicable.
